# Nasal Epithelial Barrier Integrity and Tight Junctions Disruption in Allergic Rhinitis: Overview and Pathogenic Insights

**DOI:** 10.3389/fimmu.2021.663626

**Published:** 2021-05-21

**Authors:** Siti Muhamad Nur Husna, Hern-Tze Tina Tan, Norasnieda Md Shukri, Noor Suryani Mohd Ashari, Kah Keng Wong

**Affiliations:** ^1^Department of Immunology, School of Medical Sciences Malaysia, Universiti Sains Malaysia, Kubang Kerian, Malaysia; ^2^Hospital Universiti Sains Malaysia, Kubang Kerian, Malaysia; ^3^Department of Otorhinolaryngology, Head and Neck Surgery, School of Medical Sciences, Universiti Sains Malaysia, Kubang Kerian, Malaysia

**Keywords:** allergic rhinitis, tight junction, TSLP, IL-25, IL-33, innate lymphoid cells, Th2 cytokines, epigenetic

## Abstract

Allergic rhinitis (AR) is a common disorder affecting up to 40% of the population worldwide and it usually persists throughout life. Nasal epithelial barrier constitutes the first line of defense against invasion of harmful pathogens or aeroallergens. Cell junctions comprising of tight junctions (TJs), adherens junctions, desmosomes and hemidesmosomes form the nasal epithelial barrier. Impairment of TJ molecules plays causative roles in the pathogenesis of AR. In this review, we describe and discuss the components of TJs and their disruption leading to development of AR, as well as regulation of TJs expression by epigenetic changes, neuro-immune interaction, epithelial-derived cytokines (thymic stromal lymphopoietin, IL-25 and IL-33), T helper 2 (Th2) cytokines (IL-4, IL-5, IL-6 and IL-13) and innate lymphoid cells. These growing evidence support the development of novel therapeutic approaches to restore nasal epithelial TJs expression in AR patients.

## Introduction

Allergic rhinitis (AR) is a global health problem affecting 10–40% of the population worldwide and it usually persists throughout life (1, 2). AR is a symptomatic disorder of the nose induced by an immunoglobulin E (IgE)-mediated inflammation after allergen exposure of the membranes lining the nose, and it is usually accompanied by classical symptoms such as nasal itching, sneezing, rhinorrhea, and nasal congestion (1, 3, 4). Epithelial cells play important roles as physical barrier to prevent the entry of allergens, pathogens and other foreign particles (5). Tight junctions (TJs) comprise of cell-cell adhesion complexes between epithelial cells required for epithelial barrier function. Previous studies have reported that impairment of nasal epithelial is one of the underlying causes of AR (6, 7). Differential expression of TJ molecules has also been found in distinct inflammatory phenotypes of allergic airway inflammation in mice compared to controls (8). These findings suggest that TJs disruption plays causative roles in the development of allergy through increased exposure of nasal tissues to environmental allergens (9). In this review, we present and discuss the association of TJs disruption in AR, as well as regulation of TJs expression by epigenetic changes, neuro-immune interaction, epithelium-derived cytokines, T helper 2 (Th2) cytokines and innate lymphoid cells (ILCs).

## Nasal Epithelial Barrier and Tight Junctions

### Nasal Epithelial Barrier

Epithelial barrier is the first line of defense where an intact mucosal barrier is crucial in protecting the host immune system from the exposure of harmful pathogens (10). This epithelium also plays vital roles in regulating both innate and adaptive mucosal immunity through activation of functional molecules (*e.g.* pro-inflammatory cytokines, growth factors and chemokines) (11). Epithelial cells also secrete antimicrobial substances known as antimicrobial peptides (AMPs) such as lysozyme, defensins (α and β), lactoferrin and S-100 proteins, and they are essential in the first line defense to hinder the entry of pathogens (12, 13). Mucociliary clearance conducted by ciliated epithelial cells involves trapping of microbes and microparticles in mucus layer secreted by glands, goblet cells or ciliated cells from the nasal cavity into the esophagus (14).

Apical junctional complexes (AJCs) connect epithelial cells to one another and they consist of TJs, adherens junction, desmosomes and hemidesmosomes (15). TJs are the most apically located epithelial junctions composed of over 40 proteins either as transmembrane proteins or cytoplasmic actin-binding proteins (16). TJs function in regulating homeostasis of ions, water and certain macromolecules (17, 18). Thus, TJs are crucial in producing rate-limiting barrier to inhaled pathogens. Adherens junctions are essential for cell adhesion (19), cell proliferation and differentiation (16). Desmosomes are in close connectivity with adherens junctions (10), and they play key roles in maintaining intercellular cohesion and cellular integrity (20, 21). Lastly, hemidesmosomes are responsible to facilitate the stable adhesion of the basal epithelial cells to the basement membrane (22–24), and to link the extracellular matrix to the intermediate filaments of the actin cytoskeleton.

### Tight Junctions (TJs)

TJs in the epithelial cells consist of three primary constituents of transmembrane proteins namely occludin (OCLN), claudin (CLDN) and junctional adhesion molecules (JAMs) (25). OCLN (~65 kDa) is the first identified integral membrane protein present in both epithelial and endothelial cells (26, 27). OCLN has two extracellular loops, N- and C-terminal cytoplasmic domains. The C-terminal is important for the barrier formation of TJs which it directly interacts with zonula occludens-1 (ZO-1), and the N-terminus is involved in the regulation of paracellular permeability (5).

The CLDN family contains more than 25 protein members (28). CLDNs are four-transmembrane spanning proteins consisting of a short cytoplasmic N-terminal, two extracellular loops and a C-terminal cytoplasmic domain. CLDNs serve as cellular gate to restrict the elements to pass through the epithelial (5, 10). The C-terminal of CLDNs is vital for stability and interactions with ZO-1 (28). CLDNs can be divided into two groups based on their effects on airway epithelial barrier namely the pore- and barrier-forming CLDNs (10). Pore-forming CLDNs comprise of CLDN2, CLDN10, CLDN10b and CLDN17, while barrier-forming CLDNs are CLDN1, CLDN3, CLDN4 and CLDN7 (29, 30). The unique functional groups and co-expression of CLDN family members are thought to determine the selectivity and build the strength of TJs (30).

The JAM family of proteins comprise of classical JAMs (JAM-A, JAM-B and JAM-C) and four related proteins (JAM-4, JAML, CAR and ESAM). JAMs are type I transmembrane glycoproteins composed of two immunoglobulin-like domains *i.e.* one transmembrane domain and one cytoplasmic tail of variable length containing a PDZ domain that can interact with ZO (31). They have a significant role in the regulation of cell polarity and endothelium permeability (31).

## Disruption of Tight Junctions in AR

### Occludin

Breakdown of nasal epithelial integrity is attributable to reduced expression of TJ molecules, and both OCLN mRNA and protein expressions are decreased in the nasal epithelium of AR patients compared to controls (32). Nasal epithelial barrier function has been known to be impaired in HDM-induced AR patients (33). Lower *OCLN* mRNA expression also occurred in HDM-induced AR patients compared to non-allergic controls (33, 34). Moreover, reduced ordered arrangement of OCLN was found in AR patients and the defect might expedite the passage of allergens and environmental pro-inflammatory agents through nasal epithelial barrier (33). This was demonstrated by reduced trans-tissue resistance and increased passage to fluorescein isothiocyanate (FITC)-dextran 4 kDa (FD4) in the tissues.

A lower mRNA expression of *OCLN* was present in the nasal biopsies of AR patients compared to healthy subjects and idiopathic rhinitis (IR) patients (7). This was supported by immunofluorescent imaging of OCLN from paraffin-embedded mucosal biopsy specimens of AR patients which displayed a severely disrupted layer and irregular pattern of OCLN expression compared to controls (7). *OCLN* expression also showed a decreased pattern in the mouse model of HDM-induced allergic airway inflammation with increased FD4 levels and bronchoalveolar lavage (BAL) albumin levels (7). Furthermore, HLA-DR- and CD11c-positive dendritic cells (DCs) penetrated beyond OCLN in the epithelium of the nasal mucosa of AR patients (35). OCLN is found at the uppermost layer of pseudostratified columnar epithelium of the nasal mucosa. Hence, these results indicated that DCs may gain access to antigens beyond epithelial TJs in the human nasal mucosa of AR.

### Claudins

Previous studies have shown the defect in epithelial barrier due to decreased expression of CLDNs that contribute to AR in both patients and animal models. Recently, we demonstrated a decreased expression of *CLDN3* and *CLDN7* in nasal epithelial cells (NECs) of HDM-induced AR patients compared to non-allergic controls (34). CLDN1, CLDN3, CLDN7 and CLDN12 mRNA and protein expressions were significantly decreased in nasal epithelium of AR patients compared to controls subject (32). In pollen-induced AR mouse model, mice were sensitized and nasally challenged with Japanese cheddar (JC) or ragweed pollens with or without recombinant human (rh) Cystatin SN (an endogenous cysteine protease inhibitor) to investigate the effects of rhCystatin SN on the epithelial barrier *in vivo* (36). *CLDN1* expression in the nasal mucosa was reduced in JC and ragweed-challenged mice, and only *CLDN1* expression in rhCystatin SN-treated-JC-challenged mice was maintained (36). This indicates that Cystatin SN specifically inhibits JC-induced but not ragweed-induced nasal TJ disruption through inhibition of protease activities.

In AR patients, the mRNA expression of *CLDN1* and *CLDN4* in mucosal biopsies of AR patients was decreased compared to the healthy controls (7). Lower mRNA expression of *CLDN1*, *CLDN4*, *CLDN7*, *CLDN8*, *CLDN12*, *CLDN13* and *CLDN14* were also detected in the nasal mucosa of AR patients (35). HLA-DR^+^ and CD11c^+^ DCs expressed *CLDN1* and invaded beyond OCLN in the epithelium of the nasal mucosa with AR but not in subjects without AR (35). These findings demonstrate that antigens beyond epithelial TJ in the human nasal mucosa of AR can be accessed by DCs (35). Downregulation of CLDN4 protein expression in AR nasal epithelium has also been observed by immunohistochemical staining (37).

## Environmental Factors and Disruption of TJS in AR

### Air Pollution and Disruption of TJs in AR

Environmental factors such as urban locations, air pollutants and presence of airborne allergens play contributive roles to the disruption of TJs in AR. We recently demonstrated that *OCLN* and *CLDN7* mRNA expressions were significantly reduced in AR patients sensitized to HDMs compared with non-allergic controls, and lower *OCLN* or *CLDN7* expression was associated with urban locations or exposure to second-hand smoke, respectively (34). This is comparable with recent findings that air pollution represents a risk factor for AR onset whereby epithelial barrier can be negatively affected by air pollutant such as diesel exhaust particles (DEPs) and fine particulate matter ≤2.5 μm (PM2.5) causing sinonasal diseases (38–41).

AR rat exposed to PM2.5 had significantly increased AR symptoms (*i.e.* number of sneezes and nasal rubs) and exhibited disorderedly arrangement of nasal mucosa epithelium (42), and a substantial increase in goblet cell hyperplasia and collagen deposition were also observed compared with control rats (43). The exposure of DEPs to primary nasal epithelial cells (PNECs) in air liquid interface (ALI) culture and exposure of PM2.5 to human nasal epithelial cell (HNEC) line (RPMI 2650 cells) significantly reduced the expression of TJ molecules including *OCLN, ZO-1* and *CLDN1* (38–40). It also reduced the transepithelial resistance (TER) of the cells and increased their permeability as monitored by fluorescently-labeled dextran permeability in both studies (38, 39). DEP disrupted TJs through a reactive oxygen species (ROS)‐mediated pathway, leading to increased permeability of NECs (40).

ROS suppresses TJ proteins expression through p38 MAPK, p65 NF-κB, and Akt signaling pathways independent of IL-8 and through inhibition of tyrosine phosphatase (44, 45). Cultured human epithelial cells exposed to urban PM (UPM) showed a decrease in cell viability *i.e.* detached and shrunken epithelial cells with condensed nuclei (44, 46). The exposed cell demonstrated a significantly amplified ROS pathway leading to decreased ZO-1, OCLN, CLDN1 and E-cadherin expression compared to unexposed cells (44, 46).

Treatment of cultured human epithelial cells with ROS scavenger N-acetylcysteine (NAC) or Akt inhibitor (MK-2206) reversed the effects of UPM. Through Akt inhibition, it decreased UPM-induced ROS formation and p38 and p65 protein phosphorylation, and restored the expression of ZO-1 and E-cadherin (44). Meanwhile, the mucosal epithelium was observed to be arranged in a more orderly manner when treated with ursolic acid (UA) (a pentacyclic triterpene extract from natural plants) in AR model exposed to PM2.5 (42), and decrease in number and size of goblet cells in epithelial layer, expression of MUC5AC and proportion of collagen deposition areas (43). UA possesses anti-inflammatory, antioxidant and anti-fibrotic properties where it is capable of modulating the MAPK and NF-κB signaling pathways (47).

### House Dust Mites and Disruption of TJs in AR

HDM is the most common allergens causing allergic sensitization among AR patients and mostly affecting the 25–35 age group without significant differences in both genders (48–52). The major species of HDM causing allergy include *Dermatophagoides pteronyssinus* (*Der p*) and *Dermatophagoides farinae* (*Der f*) (53–55). HDM allergen is highly associated with the disruption of epithelial barrier where they have proteolytic activity that can cleave the epithelial TJs protein. *Der p* 1 (*i.e.* a HDM cysteine proteinase allergen) has been reported to cleave extracellular domain sites of OCLN and in CLDN1 proteins, resulted in amplified epithelial permeability that allowed the passage of *Der p* 1 through the epithelial barrier (33, 56, 57). Inhibition of the protease activity of *Der p* 1 as a therapeutic approach to reduce HDM-induced barrier dysfunction has been proposed (58). Treatment of cultured primary HNECs *in vitro* with *Der p 1* showed markedly decrease in CLDN1, resulted in significantly increased FD4 epithelial permeability (32).

Breakdown of epithelial barrier can also be caused by proteases *via* fungi exposure that facilitates the access of pathogens and directly activates immune cells (59, 60). The serine proteases in fungi such as *Alternaria* has been reported to decrease the mRNA and protein expression of TJs including *ZO-1, OCLN* and *CLDN1* in PNECs (61). Reduction in transepithelial resistance and increase of ROS was demonstrated in the study (61). Exposure of *Der p1* to HNECs line (RPMI 2650 cells) and *in vivo* AR model (rats) downregulated the expression of both mRNA and protein levels of OCLN, CLDN1, ZO-1 and JAM-A as well as increased TER and FD4 permeability compared to control group (62).

## Epithelial Cell-derived Cytokines in AR

The onset of AR is also triggered by disrupted sinonasal epithelium through production of the inflammatory epithelium-derived cytokines TSLP, IL-25 and IL-33. These cytokines are key regulatory factors that connect epithelial–mesenchymal communications and elicit pathological modifications in the airway (63). Receptors expressed on the surface of epithelial cells such as Toll-like receptors (TLRs) and nucleotide-binding oligomerization domain (NOD)-like receptors (NLRs) have the ability to identify structurally conservative pathogen-associated molecular patterns (PAMPs) in pathogens and induce innate and adaptive immune response. TSLP, IL-25 and IL-33 are important in the PAMP-TLR/NLR interaction (64), and each cytokine is released into the sinonasal environment upon exposure to allergens.

### TSLP and TJs Disruption in AR

TSLP is an IL-7-like cytokine that potently induces deregulation of Th2 responses, a hallmark feature in allergic inflammatory diseases such as asthma, AD and AR (65–67). *TSLP* mRNA expression was significantly increased in the nasal mucosa of AR patients compared with controls (68). Treatment with TSLP also rapidly enhanced the barrier function of cultured HNECs together with an increase of TJ proteins CLDN1, CLDN4, CLDN7 and OCLN (68). The nasal epithelial-derived TSLP not only activates DCs but also preserves the epithelial barrier *via* upregulation of TJ proteins during the early stage of AR (68). High expression of TSLP was found in epithelium of AR patients with recruitment and infiltration of CD11c^+^ DCs (68).

TSLP was significantly upregulated in sensitized and nasally-challenged mouse model of AR (69, 70), and the expression of TSLP was suppressed by HDACi sodium butyrate (SoB) with improvements in AR clinical symptoms (69). However, TSLP expression was abolished in mast cell-deficient WBB6F1-W/Wv mice and Fc receptor γ chain (FcγR)-deficient mice (where the IgE receptor FcϵRI was not present on mast cells in these mice) compared to controls (70). This suggests that direct stimulation of epithelial cells by antigens alone may not be sufficient to induce TSLP expression in the nasal epithelium, and epithelial TSLP expression is regulated by mast cells *via* FcϵRI.

### IL-25, IL-33, Innate Lymphoid Cells (ILCs) and TJs Dysfunction in AR

Both of the epithelial-derived cytokines IL-25 and IL-33 are pro-inflammatory. IL-25, also known as IL-17E, was first identified as a Th2 cell-derived cytokine (71). The interaction between IL-25 and IL-17RA/B leads to the activation and upregulation of transcription factors (*e.g.* NFκB, STAT6, GATA3, and NFATC1). This results in the activation and polarization of memory Th2 cells, leading to the secretion of Th2 cytokines such as IL-4, IL-5, and IL-13 (63). Increased production of IL-25 was induced by dsRNA in HNECs of AR patients (72). IL-33 is most likely released through cell necrosis or injury in mucosal epithelial cells (73), and IL-33 plays central roles in the initiation of Th2 cytokines and chemokines responses in AR. IL-33 expression was increased in the serum of AR patients and IL-33 single nucleotide polymorphism (SNP) occurs in AR patients (74). IL-33 knockout murine model of ragweed pollen-specific AR showed a decrease in eosinophil accumulation, reduced ability to mount an IgE response, as well as declined expression of Th2 cytokines compared to controls. Importantly, ragweed-immunized IL-33 knockout mice showed a significant reduction in the frequency of sneezing (75).

ILCs are immune cells of lymphoid lineage where one of their main functions involves the protection of mucosal barrier (76). ILCs play important roles in the development and progression of allergic diseases including AR (77). Type 2 ILC (ILC2)-mediated immune microenvironment are characterized by the production of both IL-25 and IL-33 as well as other cytokines (*e.g.* IL-13), and presence of mast cells and histiocytes. ILCs especially ILC2s are enriched in barrier tissues such as the skin, lung, and intestine and where they regulate immune responses surrounding the epithelium and sensitization to allergens (78).

Increased IL-13-producing ILC2s were found in the blood of patients with HDM allergy, cat-sensitized adults and in grass allergic patients and in the nasal fluid upon grass pollen challenge (79–81). IL-33 is thought to be a major cytokine that mainly activates ILC2s and signifies the rationale to utilize IL-33-induced lung inflammation models (82). Recently, it has been demonstrated that ILC2s facilitate bronchial epithelial barrier disruption *via* downregulation of the TJ barrier proteins (*i.e.* OCLN) through IL-13 in asthma (82). However, the influence of ILC2s on the nasal epithelial barrier has not been examined in AR and it warrants future investigations. For further information on the roles of TSLP, IL-25 and IL-33 in innate and adaptive immune responses, reviews from Hong and colleagues (63), and Hammad and Lambrect (83) are recommended.

## Th2 Cytokines in AR

Th2 cytokines not only enhance inflammatory cell activation but also regulate epithelial cell barrier in allergic disease (*i.e.* AR, AD [atopic dermatitis], eosinophilic esophagitis, asthma and chronic rhinosinusitis) by reducing expression of TJs in epithelial cells (7, 10, 65, 84–86). The cytokines may also be released within the sinonasal microenvironment including sinonasal epithelial cells, altering TJs composition resulting in the “tight” barrier properties of TJ proteins switched to “leaky” properties (87, 88).

IL-4 is the paradigmatic cytokine involved in type-2 immune responses and plays a critical role in the development of Th2 cells and subsequent allergic reactions. Treatment with anti- interleukin-4 receptor α (IL-4Rα) monoclonal antibodies to nasal secretion of AR patients successfully restored the Th2-induced epithelial barrier dysfunction (7). It was supported by *in vivo* findings where anti-IL-4 treatment in HDM-challenged mice prevented the loss of *OCLN* and *ZO-1* mRNA expression (7). IL-4 was found to disrupt epithelial integrity *in vitro* in PNECs with reduced ZO-1 and OCLN expression (33).

Therapeutic effects of the HDACi SoB on mice with AR and Trichostatin A (TSA) on nasal lavage fluid of AR patients resulted in significantly decreased serum levels of IL-4 and IL-10, and IL-4 and IL-5, respectively (69, 89). Both inhibitors further improved clinical symptoms and SoB enhanced the nasal mucosa epithelial morphology in AR mice model (69, 89). IL-4, IL-5 and IL-13 were significantly higher in the BAL fluid of HDM-induced mice, and treatment with HDACi (JNJ-26481585) significantly reduced the interleukins to levels in non-allergic saline control mice (90). In terms of IL-6, *IL6* SNP (rs1800795) was linked with an increased risk of AR (91) and positively associated with the AR severity (92). The production of IL-6 by cultured NECs of AR was stimulated by alarmin protein high-mobility group box 1 (HMGB1), and anti-toll-like receptor 4 (anti-TLR4) blocking antibody significantly inhibited HMGB1-induced secretion of IL-6 (93).

Addition of IL-4 and IL-13 to reconstructed human epidermis cells resulted in downregulation of CLDN1 expression (85). Gene expression of *IL13* was detected in the epithelial compartment of the nasal mucosa of perennial AR patients but not observed in normal subjects (94). IL-13 was regulated by microRNA (*miR*)*-143* where overexpression of *miR-143* significantly decreased the expression of pro-inflammatory factors (*e.g.* eotaxin and mucin) responsible for producing nasal symptoms (95). Taken together, these suggest that local nasal provocation is attributable to systemic overproduction of Th2 cytokines.

## Neuroimmune and Epithelial Interaction in AR

Pathophysiology of allergic diseases involves a reciprocal regulation between neural and immune systems where both systems work synergistically to detect and respond to harmful stimuli. Neuronal cell types are usually found at skin and mucosal barrier surfaces, forming neuronal‐immune cell network (96, 97), and have been shown to regulate mucosal immunity and mucosal barrier integrity (98, 99). Neurogenic inflammation activates local release of neuropeptides and neurotransmitters such as substance P (SP), neurokinin A, neuromedin U (NMU), calcitonin gene-related peptide (CGRP), acetylcholine and norepinephrine upon activation of sensory nerve endings. This includes regulation of itch, cough, sneezing, bronchoconstriction vasodilation, plasma extravasation, recruitment of leukocytes and degranulation of mast cells (100–102).

CGRP and SP are increased in the airways of AR patients (103) and nasal secretions of idiopathic rhinitis (IR) patients (104), respectively. Neuronal ILC2s that selectively express NMU receptor 1 (NMUR1) are pro-inflammatory when exposed to NMU, and NMU is increased in the presence of IL‐25, IL‐33, and TSLP (105). In particular, NMU could activate ILC2s, and co-administration of NMU with IL-25 amplifies allergic inflammation (105).

Solitary chemosensory cells (SCCs) are found in healthy sinus cavity and turbinate tissues of the human upper airway where they function as epithelial sentinels by detecting pathogenic metabolites and initiate protective immune defense (106). Increased number of SCCs was found in the sinonasal epithelia of chronic rhinosinusitis with nasal polyps patients (107). SCCs include tuft cells (*i.e.* bottle-shaped with apical microvilli), and they are found in the nose, trachea and proximal airways. Tuft cells have close proximity with nerve fibre and can promote protective respiratory reflexes such as sneezing, release neurotransmitters (such as acetylcholine), eicosanoids and cytokines (*e.g.* IL-25 and TSLP) (108, 109). Tuft cell-derived IL-25 can initiate type-2 immune responses by activating IL-13 production in ILC2s. SCCs also utilize bitter taste receptors and canonical taste transduction pathways that play crucial roles as sentinels of respiration (110).

Neuronal transient receptor potential (TRPs) expressed in epithelial cells and endothelial cells are involved in inflammatory process and have been shown to regulate the permeability of cellular barriers in several tissues (111–113). TRP vanilloid (TRPV) channels are a group of non-selective cation channels associated with the transmission of sensory information (114). Overexpression of TRPV1 in nasal secretions were observed in IR patients (104). In the nose, nerve growth factor secreted by eosinophils can sensitize TRPV1 and TRP ankyrin 1 (TRPA1) in sensory nerve endings resulting in increased SP content and induced dendrite sprouting (115). Seasonal AR stimulated with TRPV1 intranasally with capsaicin experienced elevated symptoms and pain (116, 117). TRPV1 also mediated acidity-induced barrier dysfunction by disrupting CLDN3 and CLDN4 in human bronchial epithelial cells (16HBE) *in vitro (*118). Hence, barrier function in AR might be disrupted by increased expression or activity of epithelial TRPV1. Moreover, increased TRPV4 expression was shown in epithelial cells of AR patients compared with normal controls and in cultured epithelial cells stimulated by Th2 cytokines (IL-4 and IL-13) (119). Decreased E-cadherin and ZO-1 expression was demonstrated in epithelial cells of AR patients exposed to HDM allergen and TRPV4 agonist (GSK1016790A), suggesting possible roles of TRPV4 in the pathogenesis of allergen-induced epithelial barrier disruption in AR (119).

## Epigenetics Changes in Regulating Epithelial Barrier in AR

In recent years, more attention has focused on multi-omics approaches such as epigenomics in developing precision medicine (101). Epigenetic alterations include DNA methylation, histone modifications and regulation of non-coding RNAs that affect gene transcription by changing the structural conformation and accessibility of genes without altering the gene sequence (120, 121). In AR, epigenetics disruption has been implicated in Th1/Th2 subsets, dendritic cells function, mast cells and macrophages activation that have been well discussed (122, 123). Recently, the role of microRNAs (miRNAs), a group of short non-coding regulatory RNAs that target mRNAs for cleavage causing translational repression, is implicated in regulating epithelial barrier of AR (124). A number of miRNAs are differentially expressed in AR and asthma patients including *miR-125b*, *miR-16*, *miR-299-5p*, *miR-126*, *miR-206* and *miR-133b* (125).

Exposure of *Der p1* to HNECs (RPMI 2650) increased *miR-125b* expression by increasing the expression and activation of CXCR4 which downregulated FoxP3 expression (62). The expression levels of OCLN, CLDN1, ZO-1, and JAM-A were significantly lower in the *miR-125b*-overexpressed group and vice versa in the *miR-125b* inhibitor group compared with the control group. The autophagy inhibitor 3-MA restored TJ proteins expression in the *miR-125b*-overexpressed group. These findings was demonstrated in both *in vitro* (RPMI 2650) and *in vivo* (rat AR model) (62). This study demonstrates that the CXCR4/*miR-125b*/FoxP3 axis may play pathogenic roles in AR development by promoting autophagy in epithelial cells leading to breakdown of the epithelial barrier.

Lastly, epigenetic regulation can occur *via* histone posttranslational modifications (PTMs) such as acetylation (123). Histone deacetylases (HDACs), an enzymes responsible for removing acetyl group from lysine residues of target proteins and block genes transcription by allowing DNA to be wrapped by histones and promote chromatin condensation (9, 126, 127). HDACs are associated with defects in epithelial barrier of AR (128). Higher expression of HDAC1 protein in nasal epithelium of AR patients was associated with disruption of CLDN4 (129). Treatment with HDAC inhibitor (HDACi) JNJ-26481585 successful restored ZO molecules structure in NECs of AR patients (90).

## Epigenetic Memory

Memory of previous exposure to inflammatory stimuli is not exclusive to the hematopoietic lineage. Upon exposure to inflammatory stimuli, epithelial cells sense the signals such as from microbe-associated molecular pattern (MAMP) and transmit the information to immune cells which then facilitate chromatin remodeling of epithelial cells, resulting in increased chromatin accessibility while embedding a memory of the experience within their chromatin (130). This novel function of epithelial cells is known as epigenetic memory where epigenetic priming of enhancer regions induce stronger responses to secondary stimuli and strengthen their sensitivity towards future encounter (131).

Naik et al. demonstrated a prolonged memory to acute inflammation in mouse enable skin epithelial stem cells (EpSCs) to hasten barrier restoration after subsequent tissue damage (132). Accelerated wound healing process was observed in mice exposed to various inflammatory stimuli (*e.g.* imiquimod, abrasion wounding or *Candida* infection) compared to naïve mice, even 180 days later. This was possible because at 30 days after the exposure, most chromatin changes were still maintained in EpSCs and increased chromatin accessibility in genes encoding molecules involved in inflammation, interleukin signaling, oxidative stress response and proliferation (132).

The treatment with an anti-IL4Rα monoclonal antibody (mAb) targeting IL-4 and IL-13 receptors in patients with polyp showed preservation of several disease-associated genes including CTNNB1, a key WNT mediator that influences basal cell proliferation and differentiation (133). This explained how anti-IL4Rα mAb was capable in reducing nasal polyp burden overtime. Meanwhile, tissue-resident memory (T_RM_) cells could be maintained in barrier tissues for prolonged periods, suggesting their roles in preserving epithelial barrier (134). It was found that 80% of the transcriptomes in lung epithelial cells were dependent on CD4^+^ T_RM_ T cells including eosinophil infiltration during host defense in pneumonia and maintained even when T cells were depleted (135). CD8^+^ T_RM_ cells were also found to be maintained in the lung airways independently of tissue-circulating effector memory T (T_EM_) cells *via* a homeostatic proliferation mechanism (136).

**Figure 1:**
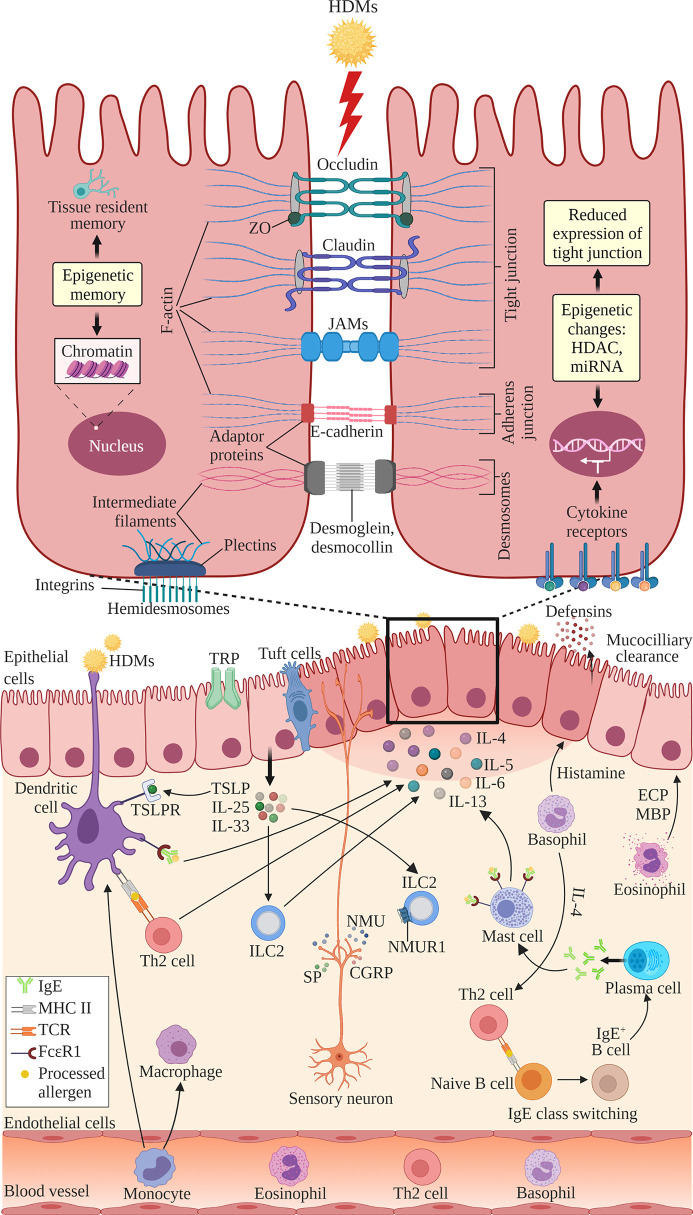
The structure of nasal epithelial barrier comprises of tight junction (TJ), adherens junction, desmosomes and hemidesmosomes. TJs are composed of occludin, claudin and JAMs that span the intercellular space and intracellular adaptor proteins ZO. Adherens junctions are composed of E-cadherins and adaptor proteins. Desmosomes consist of desmoglein and desmocollin proteins that bind internal adaptor proteins. F-actin and intermediate filaments act as cytoskeleton for these cell junctions. Hemidesmosomes comprise of plectins that link to the intermediate filaments and integrins, the transmembrane linkers of extracellular matrix and actin cytoskeleton. Epithelial cells secrete antimicrobial substances such as defensins and conduct mucociliary clearance. Epithelial activation by airborne allergens during allergic response in AR leads to the activation of epithelial cells and release of epithelial-derived cytokines TSLP, IL-25 and IL-33. This triggers subsequent activation of ILC2s and production of Th2 cytokines such as IL-4, IL-5, IL-6 and IL-13. Released cytokines promote DCs where they present antigens and activate naïve B cells to induce IgE class switching and maturation into plasma cells, which produce IgE. Secreted IgE binds the FcϵRI receptor on submucosal mast cells, leading to the release of preformed mediators such as histamine and inflammatory cytokines. In the late allergic phase, recruited eosinophils and basophils release mediators that further contribute to AR symptoms *via* epithelial damage and microvascular leaking. Blood-derived monocytes differentiate into DCs and macrophages that promote allergic responses. The overproduction of Th2 cytokines by a variety of cells suppresses the transcription of TJ molecules causing the breakdown in the nasal epithelial barrier of AR patients. Sensory neuron release NMU, SP and CGRP upon activation at sensory nerve endings. NMUR1 expressed on ILC2 intensifies the inflammatory response in the presence of IL‐25, IL‐33, and TSLP. TRP and tuft cells also have roles in regulating epithelial barrier. Epithelial barrier is also regulated by epigenetic changes through histone modification by HDACs and miRNAs. Epithelial cells exhibit epigenetic memory by embedding the memory of previous encounters within their chromatin, and tissue-resident memory cells preserve the epithelial barrier. DC, dendritic cell; ECP, eosinophil cationic protein; MBP, major basic protein; MHC, major histocompatibility complex; IgE, immunoglobulin E; ILC2, type 2 innate lymphoid cell; JAMs, junctional adhesion molecules; TCR, T cell receptor; Th2, T helper 2; TSLP, thymic stromal lymphopoietin; TSLPR, thymic stromal lymphopoietin receptor; ZO, zonula occludens; HDAC, histone deacetylases; miR, microRNA; NMU, neuromedin U; NMUR1, neuromedin U receptor 1; SP, substance P; CGRP, calcitonin gene-related peptide; TRP, transient receptor potential. Created with BioRender.com.

The structure of nasal epithelial barrier, their associations with ILCs and regulation of TJs expression by cytokines, nerves activation and epigenetic changes, are presented in **Figure 1**. In addition, **Table 1** summarizes the TJs expression in AR patients *versus* normal subjects, or in murine AR models.

**Table 1:** TJ molecules expression in AR patients *versus* normal subjects, or in murine AR models.

TJs	Protein members	Samples	Exposure	Changes in the expression of TJs
**Occludin**	OCLN	Nasal mucosa tissue of HDM-challenged mice	Anti-IL-4 antibody	Upregulated (7)
		Human PNECs with AR	No treatment	Downregulated (33)
		Human nasal mucosa tissue of HDM-induced AR	No treatment	Downregulated (7)
		NECs of HDM-induced AR patients	No treatment	Downregulated (34)
		Normal PNECs	IL-4	Downregulated (33)
		Normal PNECs	DEPs	Downregulated (38, 40)
		Normal HNECs	PM2.5	Downregulated (44, 46)
		Normal PNECs	Fungi from *Alternaria* sp.	Downregulated (61)
		Normal HNECs	*miR-125b vs. miR-125b* inhibitor	Downregulated/upregulated (62)
**Claudins**	CLDN1	Nasal mucosa tissue of pollen-challenged mice	With rh Cystatin SN *vs.* without rh Cystatin SN	Downregulated/Unchanged (36)
		Human nasal mucosa tissue with AR	No treatment	Downregulated (7, 35)
		Normal RHECs	IL-4 and IL-13	Downregulated (85)
		Normal PNECs	DEPs	Downregulated (38, 40)
		Normal HNECs	PM2.5	Downregulated (44, 46)
		Normal HNECs/NECs of rats with AR	*Der p 1*	Downregulated (32, 62)
	CLDN4	Human nasal mucosa tissue with AR	No treatment	Downregulated (7, 35)
		HNECs with AR	No treatment	Downregulated (37)
		NECs with AR	HDAC	Downregulated (129)
	CLDN3	NECs of HDM-induced AR patients	No treatment	Downregulated (34)
	CLDN7	Human nasal mucosa tissue with AR	No treatment	Downregulated (35)
		NECs of HDM-induced AR patients	No treatment	Downregulated (34)
	CLDN8	Human nasal mucosa tissue with AR	No treatment	Downregulated (35)
	CLDN12	Human nasal mucosa tissue with AR	No treatment	Downregulated (35)
	CLDN13	Human nasal mucosa tissue with AR	No treatment	Downregulated (35)

HNECs, human nasal epithelial cells; PNECs, primary nasal epithelial cells; rh, recombinant human; RHECs, reconstructed human epidermis cells; NECs, nasal epithelial cells; HDAC, histone deacetylases; miR, microRNA; DEPs, diesel exhaust particles; PM2.5, particulate matter ≤2.5 μm; Der p 1, Dermatophagoides pteronyssinus 1.

## Conclusions and Perspectives

Impairment of nasal epithelial barrier through the loss of TJs expression contributes to the development of AR. The pro-inflammatory, epithelial-derived cytokines TSLP, IL-25 and IL-33 play critical roles in AR inflammation. These cytokines activate and expand ILC2s upon encounter with allergens which subsequently induce the release of other Th2 cytokines leading to TJs breakdown in nasal epithelial of AR patients. Thus, targeted therapy against these cytokines is a therapeutic opportunity for the disease. For instance, tezepelumab, a human monoclonal therapeutic antibody that targets TSLP, is currently being evaluated for the treatment of airway allergic diseases including asthma and AD (63). Although therapeutic inhibition of TSLP or IL-33 has not been investigated in clinical trials of AR patients, murine model studies have shown that knockout of these epithelium-derived cytokines could reduce AR manifestations (75, 137). In addition, future studies should also examine the potential disruption of other components of the cell junctions such as desmosomes in AR. In conclusion, barrier defects are the results of multiple exogenous (*e.g.* air pollution, HDMs) and endogenous (*e.g.* cytokines, neuroimmune–epithelial interaction, epigenetics) triggers trapping the epithelium in a diseased state contributing to AR development, and protection of the nasal epithelial barrier integrity through restoring TJs expression is a promising therapeutic approach for AR patients.

## Author Contributions

SMNH, HTT, NSMA and KKW conceived the manuscript. SMNH and KKW designed the manuscript, wrote and revised the manuscript. SMNH prepared the figure and table. HTT, NSMA and NMS edited and revised the manuscript. All authors have read and approved the final manuscript.

## Funding

This work was supported by Universiti Sains Malaysia (USM) grants comprising of the Research University Grant (1001/PPSP/8012349) awarded to KKW, Research University Grant (1001.PPSP.8012285) awarded to NSMA and USM Fellowship Scheme awarded to SMNH.

## Conflict of Interest

The authors declare that the research was conducted in the absence of any commercial or financial relationships that could be construed as a potential conflict of interest.
